# Supplementary figures and images for: Linear-regression-based algorithms can succeed at identifying microbial functional groups despite the nonlinearity of ecological function
Source: PLoS Comput Biol. 2024 Nov 13;20(11):e1012590. doi: 10.1371/journal.pcbi.1012590 (PMC11588209; doi:10.1371/journal.pcbi.1012590)

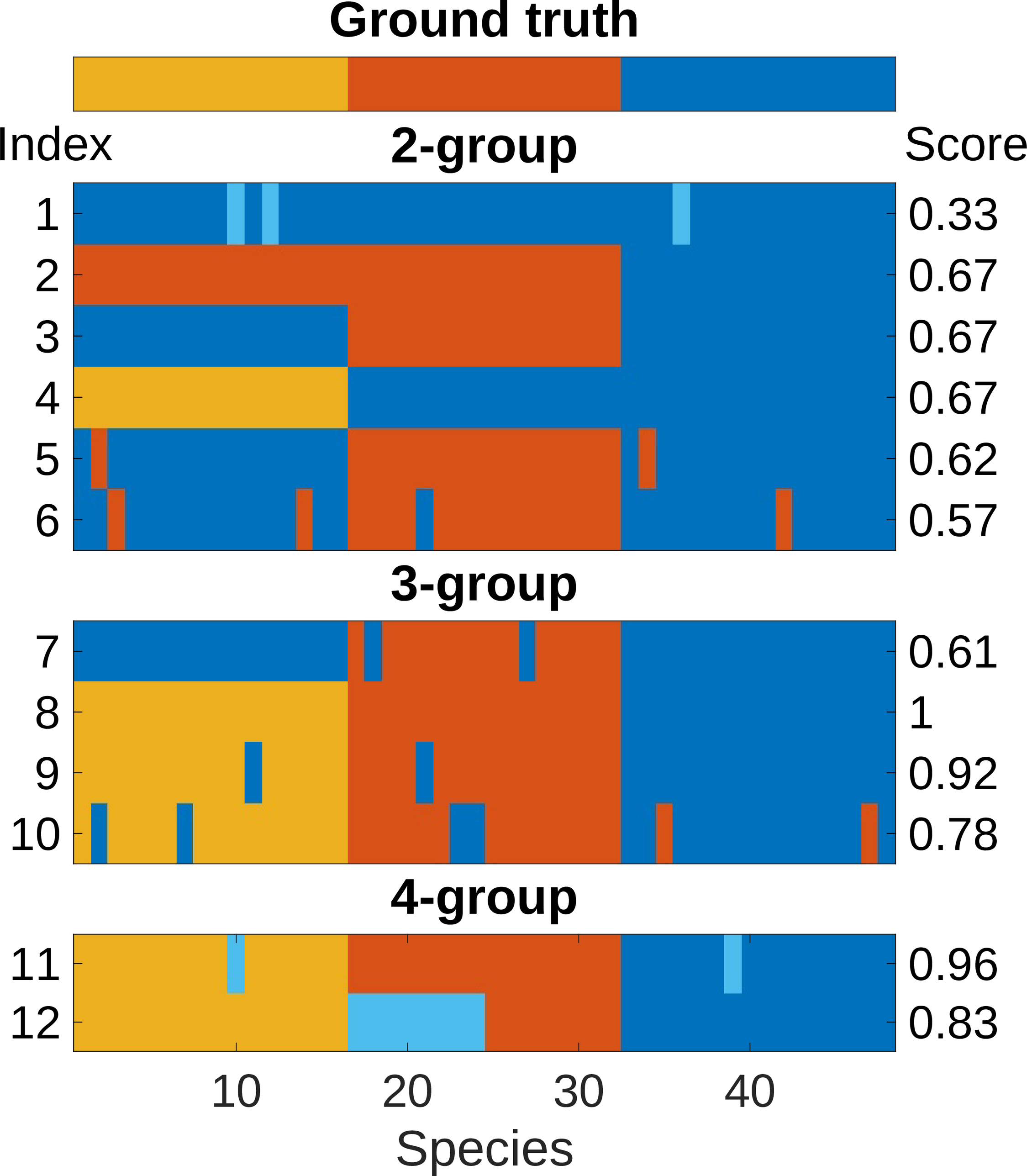

Supplement: S1 Fig — (TIF) [file pcbi.1012590.s003.tif]

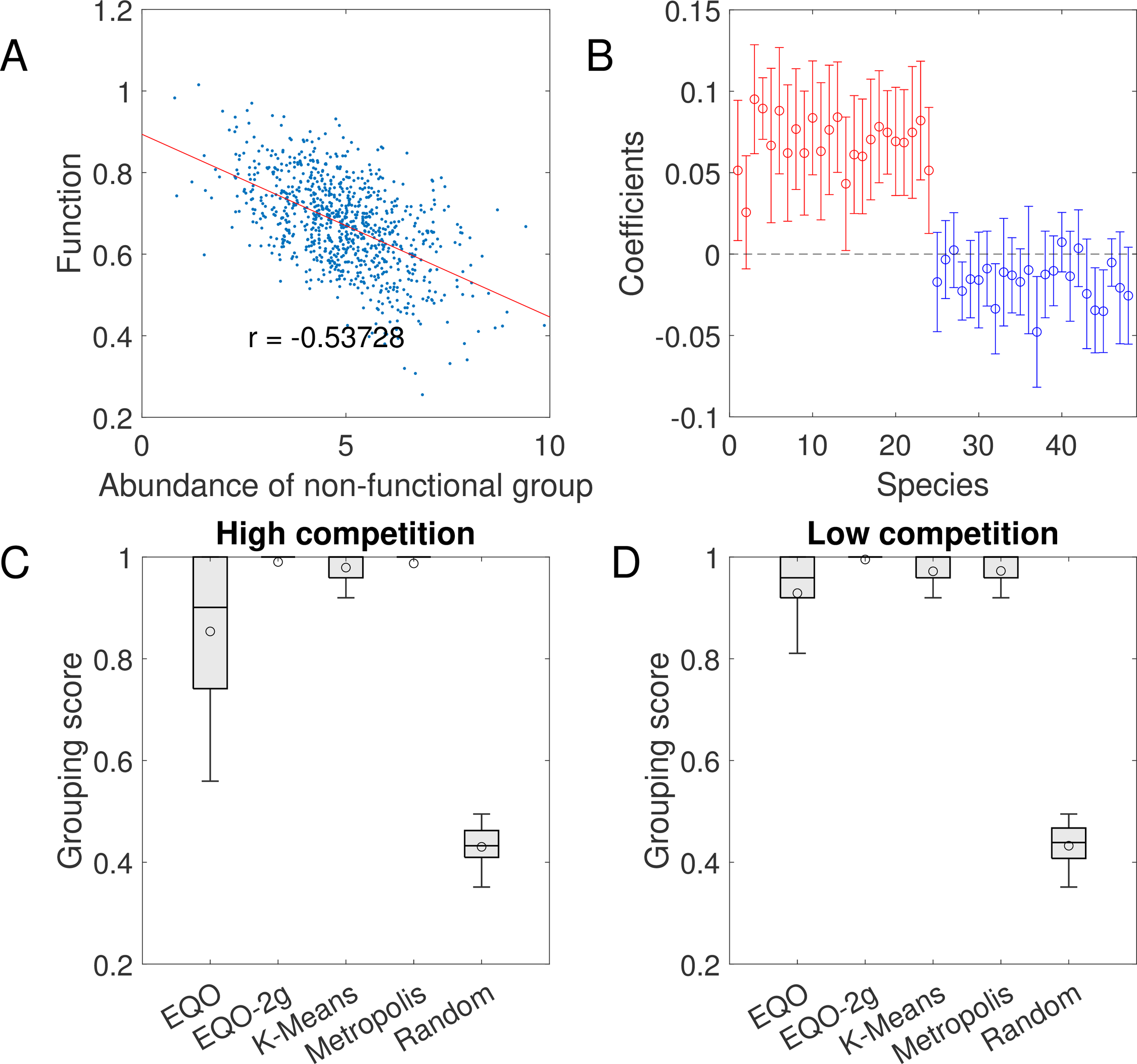

Supplement: S2 Fig — We consider the model in main text with N = 2. (A) The value of function (the final metabolite) shown against the abundance of the nonfunctional group (species not involved in producing this metabolite) for an example dataset of 900 samples. The scatter plot shows a negative correlation. The red line is the least-squares line and r marks the Pearson correlation. (B) The coefficients of all species of a S–dimensional regression of function against all S species, for the same dataset as in (A). Error bars indicate 95% confidence intervals for the coefficient estimates. The x axis is ordered so that species 1–24 belong to the functional group (red) and species 25–48 belong to the nonfunctional group (blue). We see that most nonfunctional species have negative regression coefficients. (C) The grouping scores for the outputs of EQO, EQO-2g, K-Means and Metropolis algorithms over 50 simulated datasets (see S1 Text Section 3 for EQO-2g), shown as box plot with markers the same as Fig 2A and 2B. EQO-2g performs as well as Metropolis. Random groupings are included as controls. (D) Same as (C), with the competition strength tuned down by doubling the number of general resources. The performance of EQO is improved and comparable to other algorithms, as expected. (TIF) [file pcbi.1012590.s004.tif]

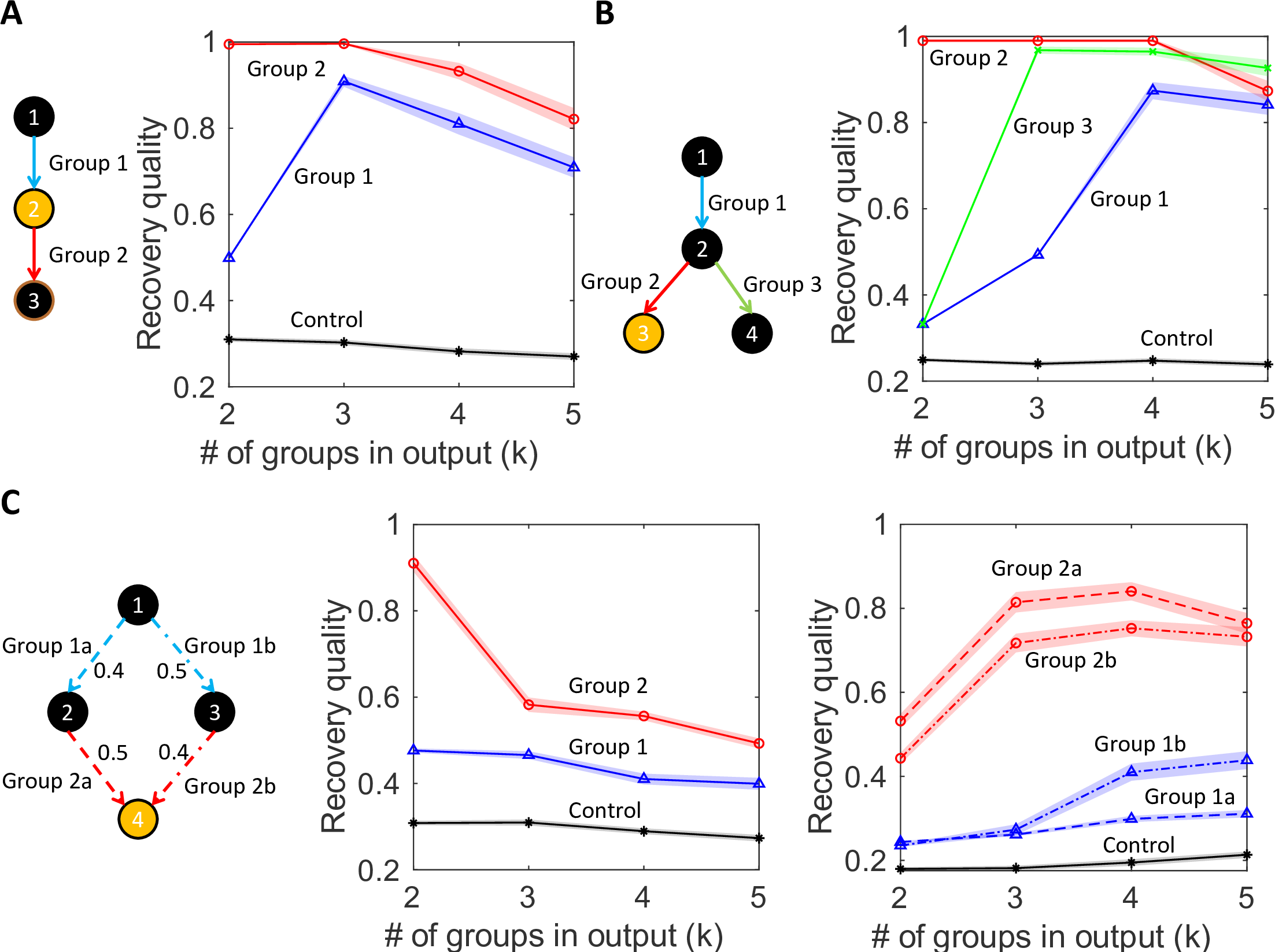

Supplement: S3 Fig — The recovery quantity of each functional group as function of number of groups in output (k) for function to be (A) intermediate product of linear degradation chain; (B) one of the end product in a degradation chain with a branch; (C) common end product of 2 linear degradation chain. Groups are indicated in the pictogram of each panel. Numbers in the pictogram of (C) indicate the transfer ratio wr of each reaction. In the first two cases (A & B), Metropolis can identify all the functional groups. While in the last, it can only recover the group of direct producers. (TIF) [file pcbi.1012590.s005.tif]

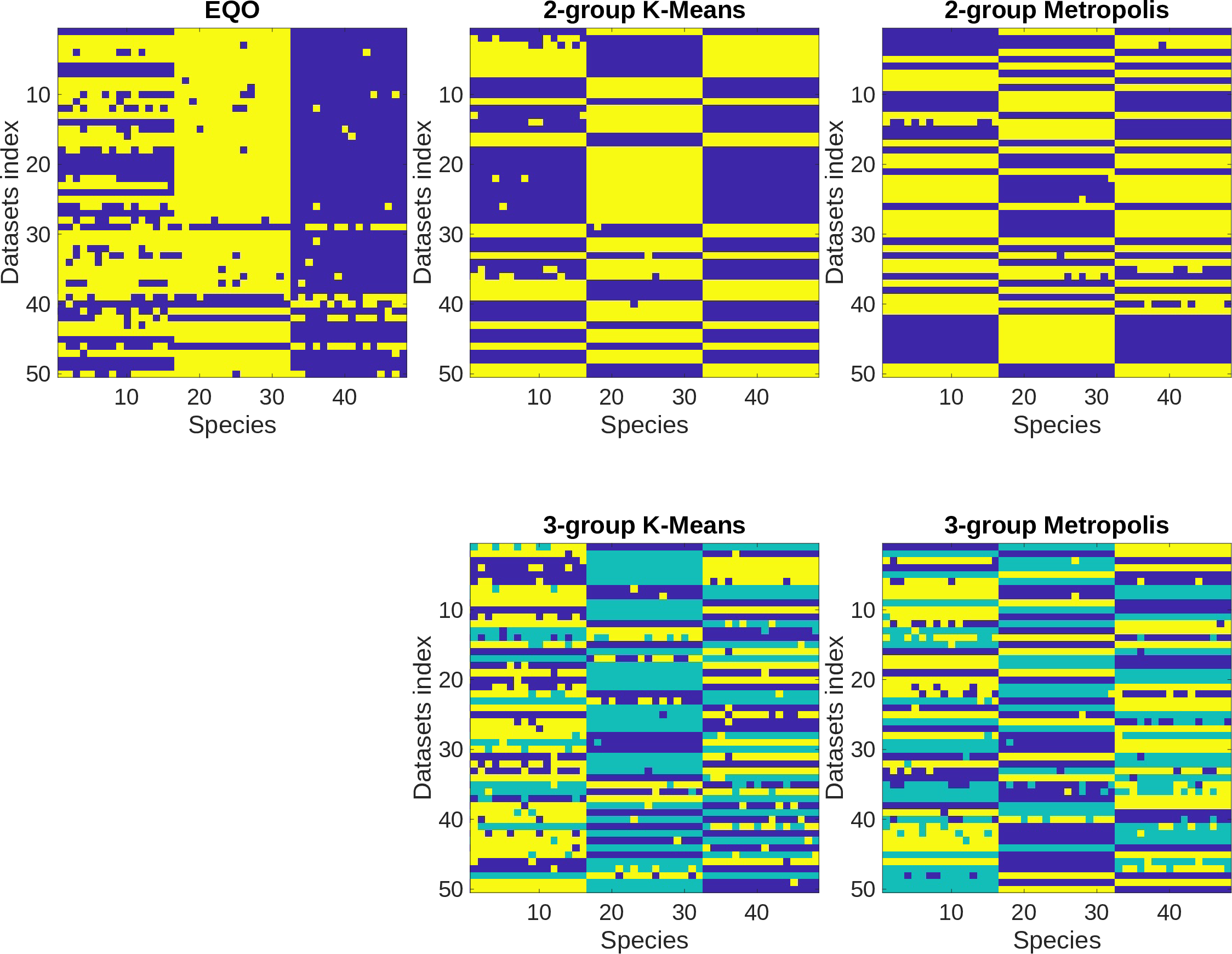

Supplement: S4 Fig — The output groupings of EQO, 2-group and 3-group K-means and Metropolis, as correspond to Fig 2A and 2B in the main text. Species 1–16 belong to group 1, 17–32 belong to group 2 (direct producers), 33–48 belong to group 3 (nonfunctional species). (TIF) [file pcbi.1012590.s006.tif]

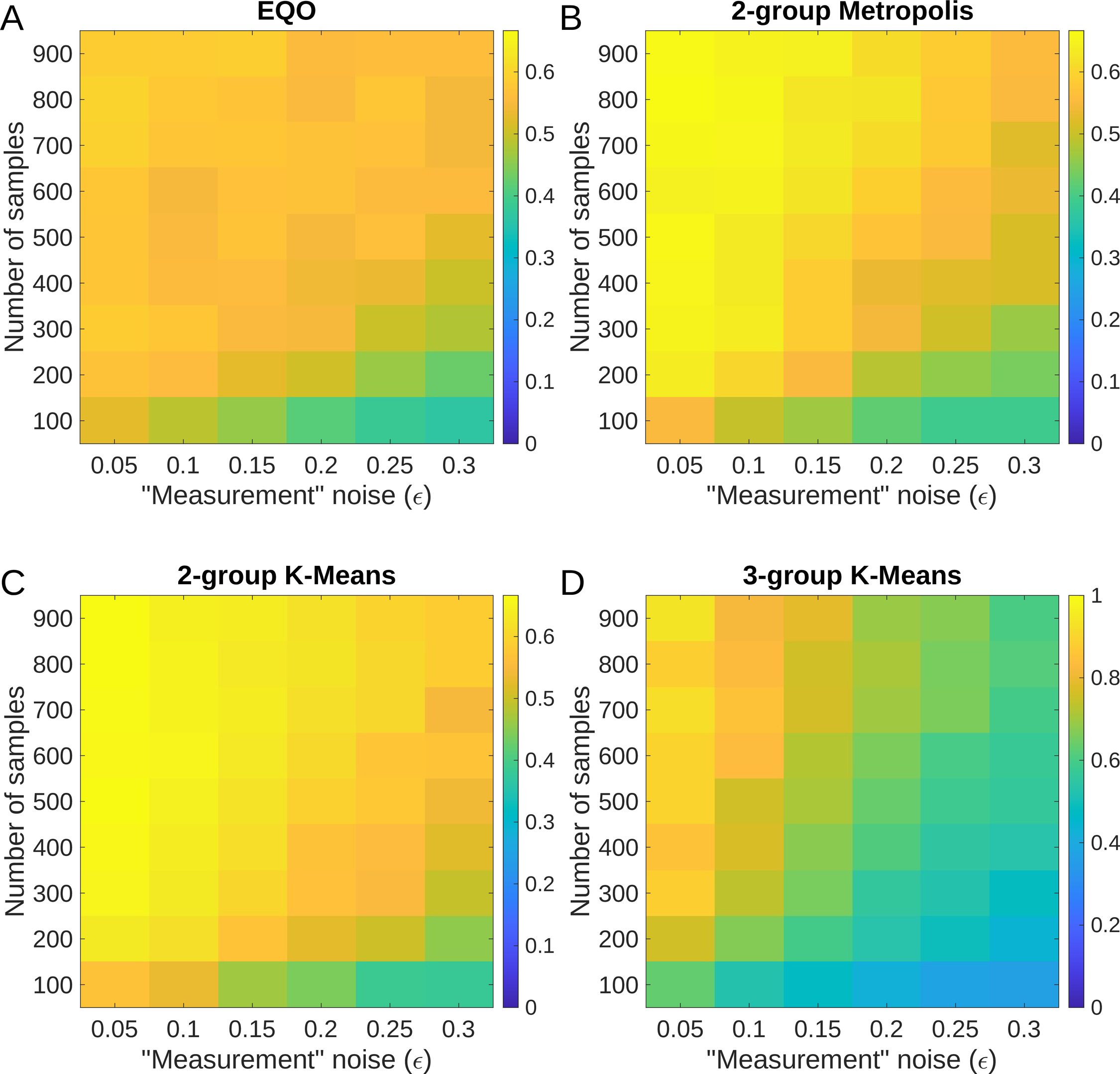

Supplement: S5 Fig — (TIF) [file pcbi.1012590.s007.tif]

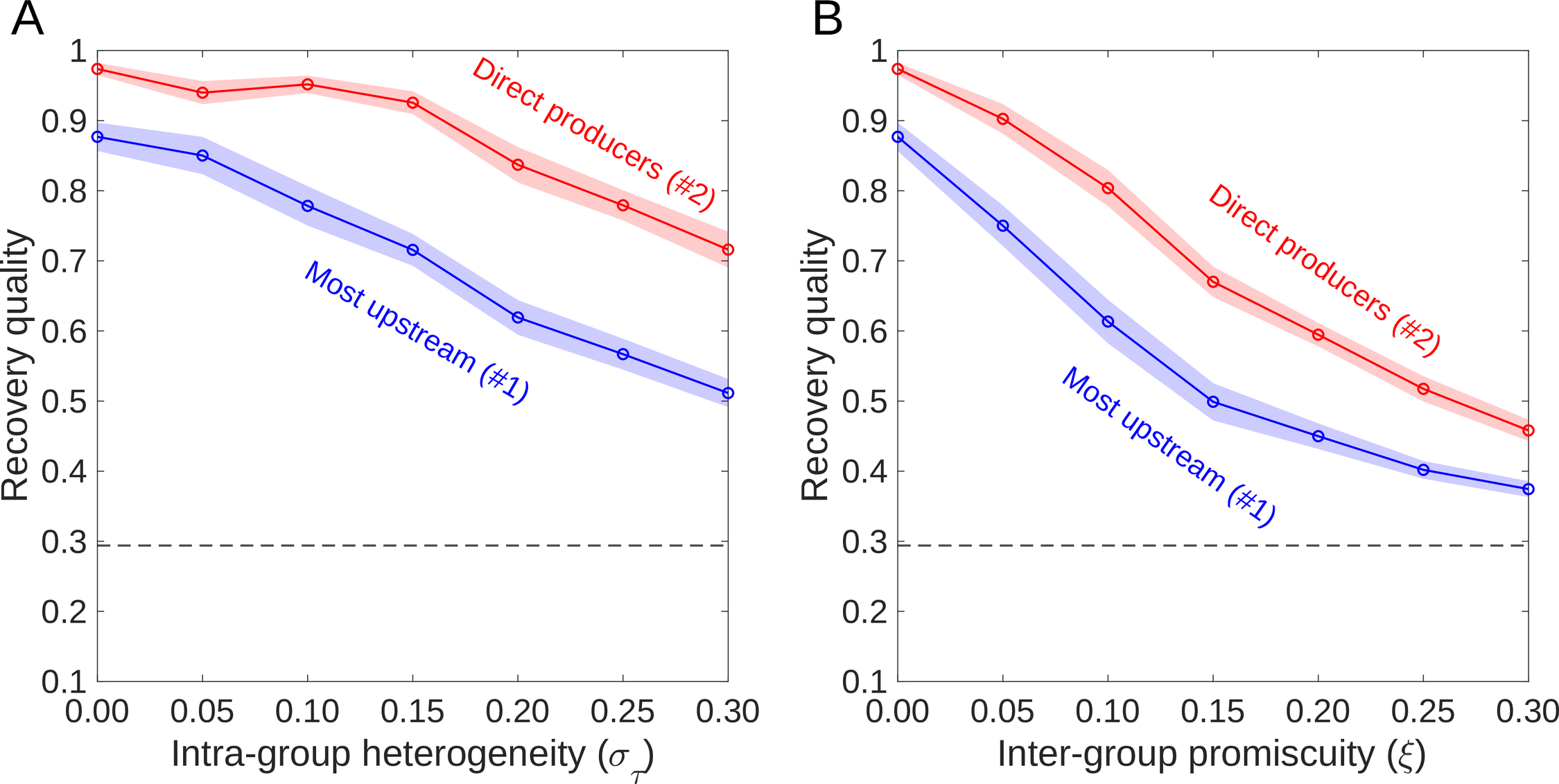

Supplement: S6 Fig — As an extension of the Fig 2D analysis in the main text, we look into the per-group recovery quality (defined in S1 Text Section 2) of the 3-group Metropolis, for the scenario of a linear degradation chain of N = 3 metabolites. (A) The recovery quality of the upstream group 1 and the direct producers group 2 shown as a function of intra-group heterogeneity (no promiscuity). (B) Same, as a function of inter-group promiscuity (no heterogeneity). Black dashed line is the random-group control (average quality of a 3-group random grouping). (TIF) [file pcbi.1012590.s008.tif]
