# Supplementary material for: Linear-regression-based algorithms can succeed at identifying microbial functional groups despite the nonlinearity of ecological function
Source: PLoS Comput Biol. 2024 Nov 13;20(11):e1012590. doi: 10.1371/journal.pcbi.1012590 (PMC11588209; doi:10.1371/journal.pcbi.1012590)
Supplement: Attachment — Submitted filename: Plos_Comp_6_2024.pdf [file pcbi.1012590.s009.pdf]

In their manuscript “Linear-regression-based algorithms can succeed at identifying microbial functional groups despite the nonlinearity of ecological function” the authors further explore the approach to functionally group microbes without using genomic information. I find this overall approach very interesting, and it makes surely sense to explore it in more depth. The authors show that several approaches that describe the community function as a linear combination of a function of microbial groups allow grouping microbes into functional groups even when the actual community function does depend on single species in a nonlinear way.

I feel the manuscript is overall very thoroughly done and well presented. I have only one major concern that resolves around the choice of the resource consumer model. I feel the way the model was chosen makes it quite likely that the approach of “grouping” based on linear regression works. The model was chosen to be a “chain type” degradation/production of a resource. However, I would intuitively (and It is not more than that, it might be wrong what I write from hereon) expect that in such a scenario the requirement of (I) complementary within a functional groups has to be fullfilled and also (II) the groupsizes have to correlate with the production rate of the endproduct. Concerning (I): If for some reason the cell number of one species in a group goes down, the educt of this group will accumulate and thus cause some other member in this group to grow stronger. So there will be anti-correlation within a group. Concerning (II): The production of N is set by the fluxes of resources through the single groups. So in order to increase the production rate of N the groupsizes have to increase, to increase fluxes. Therefore, the groupsizes correlate with the production of N.

However, I feel for a different model (like a network of resource exchange) or a situation where the function of the community depends in a more complex way on the functional groups (e.g., non-monotonic) I would expect that the approach works much less. I feel in particular when theoretical models are used to study these grouping approaches, this offers a good change to explore the limits of this grouping approach. So I would like to better understand how the choice of the model impacts the success of the grouping by linear regression and what the limitations of this approach are.
