# Supplementary material for: Linear-regression-based algorithms can succeed at identifying microbial functional groups despite the nonlinearity of ecological function
Source: PLoS Comput Biol. 2024 Nov 13;20(11):e1012590. doi: 10.1371/journal.pcbi.1012590 (PMC11588209; doi:10.1371/journal.pcbi.1012590)
Supplement: Attachment — Submitted filename: PCOMPBIOL-D-24-00586_reviewer_notes.pdf [file pcbi.1012590.s010.pdf]

Dear Editor,

We are resubmitting for your attention a revised version of our manuscript "Linear-regression-based algorithms can succeed at identifying microbial functional groups despite the nonlinearity of ecological function". We thank you for obtaining the expert reviewer reports and for your patience.

We were very happy to see that the reviewers found the work to be "very thoroughly done" (reviewer #1) "interesting and relevant for a broader audience" (reviewer #3), and were "enthusiastic about the paper" (reviewer #2). However, the reviewers also provided us with a list of suggested improvements, centered around three main themes: (1) opportunities to strengthen our analysis by assessing more ways to stress-test and "break" the method; (2) the need to better contextualize our modeling choices; and (3) several specific issues regarding presentation clarity. These and other comments have all been addressed in the revised manuscript.

Point (1) received our particular attention, with two new analyses (sensitivity of the method to "heterogeneity" and "promiscuity" of species contributions), two new supplementary figure panels, and a new figure panel in the main text. For point (2), we included additional explanations in the Introduction, Discussion, and Model sections, as well as three new references. Finally, for point (3), we subjected the explanatory Fig. 1 to a serious overhaul and made extensive clarifying edits throughout the document, detailed in the attached PDF highlighting all changes. The revised manuscript also incorporates a multitude of other improvements made in response to reviewers' suggestions, as detailed below. We believe that these changes have strengthened the manuscript substantially and would like to thank the reviewers for their careful reading and suggestions.

We include a point-by-point response to the reviewers below and provide a separate PDF file highlighting the revisions we made. Thank you for your attention to our manuscript – we look forward to hearing from you.

Sincerely,
Yuanchen Zhao,
Otto Cordero,
Mikhail Tikhonov.

Reviewer #1:
In their manuscript “Linear-regression-based algorithms can succeed at identifying microbial functional groups despite the nonlinearity of ecological function” the authors further explore the approach to functionally group microbes without using genomic information. I find this overall approach very interesting, and it makes surely sense to explore it in more depth. The authors show that several approaches that describe the community function as a linear combination of a function of microbial groups allow grouping microbes into functional groups even when the actual community function does depend on single species in a nonlinear Way. I feel the manuscript is overall very thoroughly done and well presented.

We thank the reviewer for the careful read of our manuscript, and for this positive assessment.

I have only one major concern that resolves around the choice of the resource consumer model. I feel the way the model was chosen makes it quite likely that the approach of “grouping” based on linear regression works. The model was chosen to be a “chain type” degradation/production of a resource. However, I would intuitively (and It is not more than that, it might be wrong what I write from hereon) expect that in such a scenario the requirement of (I) complementary within a functional groups has to be fullfilled and also (II) the groupsizes have to correlate with the production rate of the endproduct. Concerning (I): If for some reason the cell number of one species in a group goes down, the educt of this group will accumulate and thus cause some other member in this group to grow stronger. So there will be anti-correlation within a group. Concerning (II): The production of N is set by the fluxes of resources through the single groups. So in order to increase the production rate of N the groupsizes have to increase, to increase fluxes. Therefore, the groupsizes correlate with the production of N. However, I feel for a different model (like a network of resource exchange) or a situation where the function of the community depends in a more complex way on the functional groups (e.g., non-monotonic) I would expect that the approach works much less. I feel in particular when theoretical models are used to study these grouping approaches, this offers a good change to explore the limits of this grouping approach. So I would like to better understand how the choice of the model impacts the success of the grouping by linear regression and what the limitations of this approach are.

Thank you for the comment. The reviewer makes two points in this paragraph, which we address separately:

1. The choice of linear chain model likely favors performance.
   We agree, and this is intentional. This manuscript originated from the surprise of some of the authors that an approach like EQO, which is based on very simplistic assumptions, can work at all. Thus, our first intention was to show why such an approach can work. For this, it is convenient to start with a linear chain-like model. We revised the manuscript to state this explicitly (lines 109-110). Our second intention is to watch the method fail; which brings us to reviewer's second point:
2. For a study like ours, it is informative to challenge the method enough to "watch it fail".
   We fully agree, and this is why we indeed follow the linear chain example with exploring more complex network topologies, and observe the performance dropping.

However, we agree with the reviewer that this analysis could have been more developed. Accordingly, the revised manuscript now includes two more ways of challenging the regression-based grouping, namely making species' contributions increasingly heterogeneous rather than identical (detailed in new SI fig S6A); and increasingly promiscuous rather than specializing in a single reaction step (detailed in new SI fig S6B). We edited the Results section to make these points more prominent (lines 140-147; lines 212-228) and added a new panel to the main text Fig. 2 (new Fig. 2D). Finally, we expanded the Discussion to mention additional failure modes (lines 316-321).

The reviewer is correct that we do not consider a full-on complex network of resource exchange; the reason is that in such a situation it's not clear what grouping *should* conceivably be treated as "correct", making it difficult to score algorithm performance (lines 89-91; lines 307-309).

Looking ahead, we agree with the reviewer that the natural and crucial next step would be to identify which real-life functions are likely to be amenable to such grouping methods. However, we believe this would be best done on empirical data. We are working with an experimental collaborator who has high-quality data from natural soils, and expect to be able to say more soon. However, that would be a data-based analysis and thus lies beyond the scope of the present manuscript.

Reviewer #2:
The authors seek to define groups of organisms that perform specific subfunctions that are part of a community metabolism. They propose an approach where the organisms performing these subfunctions can be fit from data given a model. They propose a consumer-resource model where a community of microbes carries out a collective function. They create synthetic data with the model. They compare three algorithms to fit the groupings of the species based on the relative abundances and the function score. Biologically, this scenario is interesting because it provides a mechanism for community-level selection in microbial communities: if selection acts on the degradation (or synthesis) of a product, then all of the necessary species for its production are selected for as a group. Methodologically, this paper extends the EQO approach previously developed by the authors with a Metropolis algorithm-based fitting approach that correctly detects the groupings with good accuracy in the synthetic dataset. Overall, I am enthusiastic about the paper. The work appears thorough, and I have no major concerns. There were some aspects of the presentation that were a bit opaque because the authors refer to past work a lot without providing the specific pertinent details from the cited works. These instances can be improved with minor rewriting. I think the paper is a good fit for PLOS Computational Biology.

We thank the reviewer for such a warm assessment. No-major-issues reviews are an increasingly rare gift in our profession, and we are grateful.

Here are some very minor suggestions to improve the manuscript. My guess is that some of these comments are based on my own misconceptions, and I hope the authors will improve the presentation so that readers do not come away with misconceptions.

. I appreciate the brevity but feel it would be helpful to expand the introduction to briefly summarize the ensemble quotient optimization approach in a bit more detail for the naiive reader. Some of this could potentially be accomplished in Figure 1 as well.

Yes, thank you. We edited / expanded the introduction to eliminate any expectation of prior familiarity with the EQO approach (lines 55-60).

. Figure 1 is something of a graphical abstract intended to summarize the approach of the paper, but it does not make certain specifics clear enough. I wasn't sure from Fig. 1 what the model is assuming. If this is a linear pathway with intermediates, a product will not be synthesized unless all the intermediate steps are completed. Or is the idea that all enzymes in the pathway are present in each 15-member community, and it is the abundance of each of the 15 members that determines the flux through their step in the pathway? From the reading the whole paper, I assume the latter, but please clarify early on. Also, could the colors in 1a correspond to those in 1c? For instance, coloring by group in 1a could be one way to make it more consistent.

Thank you for prompting us to improve this cartoon. In retrospect, we agree the original version was indeed very confusing, so this comment received our particular attention. For this revision, we've talked several colleagues through a few iterations, and we hope you will find the new version to be a considerable improvement. In particular:
* The revised text clarifies that all steps need to be completed for the final product to be produced; line 124. (However, for a 2-step reaction chain, it is exceedingly unlikely for a reaction step to not be represented among the 15 taxa.)
* We added an explicit statement clarifying that species abundances determine fluxes (line 148).
* We edited panel A to focus on the 3-group case, which is the starting point of our analysis.
* We edited panel B to remove distractions, and to be a better visual representation of our sampling strategy.
* We flipped panel C and edited the colors to better represent how the species correspond to the 3-step pathway.
* We made other clarifying edits in the figure and legend; see document highlighting edits made.

. I found the growth function (eqn 1a) a bit unintuitive the way it is presented, and I would like to see a clarification. Here is why I think I was confused: I am used to thinking of bacteria feeding on one limiting resource at a time, in the spirit of the lac operon in E. coli. From a consumer-resource perspective, Liebig's law of the minimum makes the non-limiting resources ignorable. In eqn 1a, the growth is a summation of two resource utilization terms balanced by the maintenance cost of the species. How should I think of this in terms of setting the growth rate? Are these two independent ways the population can grow? Why isn't just the limiting resource setting the growth rate? Should we think of a time-averaged growth rate where the growth on multiple limiting resources that fluctuate over time needs to be factored in?

Yes, you are correct. The form of eqn 1a makes a fairly strong assumption of substitutable resources, an assumption we should have better contextualized. This choice, and the additive form of eqn. 1a ignoring Liebig's law, was motivated by our desire to pick the simplest model (fewest parameters, fewest choices), and the model as written, with slight variations, has been extensively used in the high-diversity ecology literature as the simplest metabolism-explicit model. The revised version clarifies this reasoning (lines 118-119; 140-147), and provides additional references (lines 119 and 141; new refs 32-34).

. In equation 1a, first summand in the brackets; equation 1b, third summand; and equation 2, first summand: it appears the efficiency of degradation of a metabolite by a species is 100%? I would think there would be a rate constant, such that a fraction of the resource is

consumed/converted. However, I also see that the realized growth is greater than 1 and there is a metabolic cost term, so it's not normalized to 1. Things probably balance out in the end. This seems like a good strategy to reduce the number of parameters to reduce overfitting. Was that the intent? Could the reasoning be explained a bit more in the text? I see references to previous papers, and these are nice papers, but explaining it a bit more here would be helpful.

Yes, the intent was again simplicity (fewer parameters). We revised to state this explicitly (line 140), and expanded the section to provide more explanations, particularly for the metabolic cost term (lines 134-139).

. Very minor: End of page 4: Please add additional citations of the Materials and Methods section to the mention of each algorithm. It wasn't clear that the descriptions were there, and they greatly help.
Good point, thank you. We added the citations as suggested (line 164 and hyperlinks on lines 165, 168, 172).

. Certain embellishments are confusing. For example in the abstract, "This success seems puzzling, since modeling community function as a linear combination of contributions of individual species appears simplistic." I'm not sure why it seems puzzling when the function is governed by a linear pathway. Can the sentence be left out? The paper seems pretty straightforward, but the writing in certain cases alludes to something more mysterious, which is confusing.
Thank you. We edited the abstract (line 21).

Reviewer #3:
This manuscript by Yuanchen Zhao, Otto Cordero and Mikhail Tikhonov applies several algorithms for functional group detection in ecological communities (including a newly proposed algorithm) to a simulated dataset of species abundances and measurements of a community-level functional trait. The authors show that several approaches, and especially their newly introduced method, can correctly recover the true functional grouping in the generated dataset, and that models based on straighforward linear associations can outperform more complex models with respect to the correct identification of functional groups. Based on these results, the authors provide important clues on the limitations of the different algorithms and their applicability in different contexts, which makes this manuscript interesting and relevant for a broader audience. However, I feel the authors could have gone a bit further in exploring the limits of applicability of functional group detection algorithms within the scope of their model. Another concern I brought up in my comments is that the assumption of near-neutrality of species within functional groups (species are interchangeable, except for their random consumption of generic resources) might have coloured the results, in particular with respect to the simultaneous detection of multiple functional groups from one-dimensional functional data. Because the manuscript for review did not have line numbers, I have included these and other remarks as comments in the attached annotated PDF.

We thank the reviewer for the careful read of our manuscript. And, please accept our sincere apologies for the oversight re line numbers! Below, we reproduce all the comments from the annotated PDF you provided.

Comments from the PDF (line numbers correspond to comment location according to the revised manuscript line numbering):

Abstract: Please specify the meaning of the abbreviation EQO.

Yes, thank you. Corrected.

Line 118: In the description of the consumer-resource model, I suggest to clarify that the resources are substitutable (in contrast to what is assumed in classical consumer-resource models of competition for essential resources)

Yes, this is an important point. Done (lines 118-119; line 140).

Eq. (1b): In combination with an unspecified binary matrix D, it is not obvious that conversion of metabolite m_j contributes an inflow of w_j to all product metabolites (if there are multiple ones). This may be addressed by specifying $D_{ij} = \delta_{i, j+1}$ from the start (note that the equations are presented in a different notation anyway in SI section 4).

Thank you for pointing this out. We aimed for generality, but in retrospect we agree that our D_{ij} notation was heavy without actually being general (and indeed, the SI section with generalizations uses a different notation anyway). We followed this suggestion and rephrased the equations using $\delta_{i, j+1}$ from the start, directly in Eq. 1b. (Lines 122-123)

Eq. (1b), continued: Another suggestion to streamline the notation is to incorporate the association between species and metabolites as a filtering of the summation index, i.e., to write $\sum_{\mu : \tau_{j\mu} = 1}$; this way, the rate expressions are easier to interpret, and the role of τ as a grouping variable is highlighted by the notation.

This is a very good point. For binary τ, as we used in the original submission, the change suggested by the reviewer would have made the equations more readable. However, in the revised manuscript we introduced new ways to challenge the method by making species contributions to function non-uniform within a group, or promiscuous between groups. This amounts to making $\tau_{j\mu}$ (contribution of species mu to reaction i) non-binary and non-sparse, at which point the notational simplification suggested here by the reviewer no longer applies. Instead, we made a different readability improvement by splitting metabolite dynamics (formerly one equation) into equations (1b) and (1c), written separately. (lines 122-123)

Line 294: Might this be because the linear model favours the inclusion of a species in a functional group if it has a consistent association with the functional property, whereas quadratic regression is vulnerable to overfitting and may erroneously favour the inclusion of species that have inconsistent effects on the functional property? In that case, one might expect linear models to be more robust predictors for detecting functional groupings that rely on fixed/non-plastic species traits

Yes; we share this intuition. (That said, we worried that adding an explicit discussion of this intuition to the text might read as too speculative: Given the purely computational nature of our study, discussing which real functions might be better suited to linear algorithms could be seen

as overreach. We prefer the more cautious “in some cases, linear **may** outperform quadratic” – which is a claim we did show…)

Line 297: Here, an important caveat is that the quality of the group prediction (grouping score) is relatively poor in that region of the parameter space (Fig 2C).

Yes; very good point. We added this caveat (lines 297-299).

The following two comments are related, encouraging us to better explore the failure modes of the group detection approach; and we address them together below (boldface added):
Line 250: This is a striking result that provokes several follow-up questions. The authors address some of them in the following paragraph. Yet, after considering these additional scenario's, I'm still wondering to what extent the ability to detect multiple groups might rely on the fact that species are almost exchangeable within each functional group in this model (except for their potentially different consumption of the generalised resources). As a result of this near-neutrality, each species within a single group contributes to the same extent, so that different groups can be distinguished based on differences between their group-specific degree of association with the functional trait. This would probably change is the model incorporated more variation between species, **such that species may belong to a functional group qualitatively, but contribute to function to quantitatively different degrees**.

Line 325, Discussion section: This is a valid point, which, in my opinion does not distract from the relevance of this study. However, I feel the authors could have done a bit more to push the proposed methods to its limits within the scope of their model. This would have provided additional insight in the robustness of the method and its vulnerabilities that must be taken into account in the application to real data. **For example, one would expect the method to perform more poorly if the generalised resources are made to have a larger impact on species growth rates (compare, for example, Fig S2C and S2D)**. If so, this would suggest that groupings closely connected to microbial fitness traits might be easier to detect than others. I hope the authors are willing to push the limits of their method along this and similar lines, and provide more insight in its ideal use-cases and limits of application.

These are excellent points, which were also raised by reviewer #1. The revised manuscript now include an analysis where we allow species to contribute to unequal degrees, as proposed by the reviewer. We also now challenge the approach along another dimension, considering species that are increasingly promiscuous rather than specializing in a single reaction step. Addressing the "**Line 325 comment**" above, the impact on the overall method performance is summarized in the new main text figure panel 2D. Addressing "**Line 250 comment**", two new supplementary panels (Fig. S6A and S6B) present the per-group analysis, showing that promiscuity and heterogeneity of species contributions impact the detection ability of both "upstream" and "direct producer" groups. We edited the Results section (lines 217-228) accordingly, and expanded the Discussion to mention additional possible failure modes (lines 316-321). We also added an explicit mention of the reviewer's point highlighted in bold (lines 212-213).

Looking ahead, we agree with the reviewer that the natural and crucial next step would be to identify which real-life functions are likely to be amenable to such grouping methods. However, we believe this would be best done on empirical data. We are working with an experimental collaborator who has high-quality data from natural soils, and expect to be able to say more

soon. However, that would be a data-based analysis and thus lies beyond the scope of the present manuscript.

Line 405: I would expect a division by beta in this formula, if beta = 0 corresponds to the zero-temperature regime, as stated below.
Thanks for catching our mistake! The zero-temperature regime is indeed beta=infinity. Fixed. (line 405)
